# Development and validation of a method for mercury determination in seawater for the process control of a candidate certified reference material

**DOI:** 10.1007/s00216-015-8833-9

**Published:** 2015-06-23

**Authors:** Raquel Sánchez, James Snell, Andrea Held, Hendrik Emons

**Affiliations:** European Commission, Joint Research Centre (JRC), Institute for Reference Materials and Measurements (IRMM), Retieseweg 111, 2440 Geel, Belgium

**Keywords:** Mercury, Water Framework Directive (WFD), Seawater, Inductively coupled plasma mass spectrometry (ICP-MS), Method validation, Uncertainty estimation

## Abstract

A simple, robust and reliable method for mercury determination in seawater matrices based on the combination of cold vapour generation and inductively coupled plasma mass spectrometry (CV-ICP-MS) and its complete in-house validation are described. The method validation covers parameters such as linearity, limit of detection (LOD), limit of quantification (LOQ), trueness, repeatability, intermediate precision and robustness. A calibration curve covering the whole working range was achieved with coefficients of determination typically higher than 0.9992. The repeatability of the method (RSD_rep_) was 0.5 %, and the intermediate precision was 2.3 % at the target mass fraction of 20 ng/kg. Moreover, the method was robust with respect to the salinity of the seawater. The limit of quantification was 2.7 ng/kg, which corresponds to 13.5 % of the target mass fraction in the future certified reference material (20 ng/kg). An uncertainty budget for the measurement of mercury in seawater has been established. The relative expanded (*k* = 2) combined uncertainty is 6 %. The performance of the validated method was demonstrated by generating results for process control and a homogeneity study for the production of a candidate certified reference material.

## Introduction

The Water Framework Directive (WFD) [1] provides a list of priority substances that present a risk for the good chemical status of the aquatic environment defined in terms of compliance with all the environmental quality standards (EQSs) established in the daughter Directive 2013/39/EU [2]. The EQS for mercury expressed as a maximum allowable concentration (MAC) is 0.07 μg/L. In addition, the Directive 2009/90/EC [3] states that laboratories performing the control shall demonstrate their competence by the analysis of reference materials that are representative of collected samples. Certified reference materials (CRMs) for trace elements in natural waters are widely available. However, there are a limited number of CRMs for mercury at or below the level of the EQSs [4]. Seawater presents a particular challenge as only one CRM is available and its concentration is 35 times lower than the established EQS. Therefore, a CRM for the determination of mercury in seawater is under development at JRC-IRMM. The target Hg mass fraction for this CRM is three times lower than the EQSs.

CRMs must be characterized using analytical methods validated according to ISO/IEC 17025 (http://www.iso.org/iso/home.html) during development and production to fulfil the requirements of ISO Guide 34 (http://www.iso.org/iso/home.html). In practice, methods are also often needed to rapidly generate results to control processing activities at the time of CRM processing. The targeted mercury mass fraction is at a level below detection limits for many techniques. Due to the high mobility of this element, there is the risk of contamination, volatilization and adsorption losses in all steps of the analysis [5]. Several studies have been carried out using spectrometric techniques for mercury determination [6–10]. Most of them are based on atomic absorption spectroscopy (AAS) [11–15], atomic fluorescence spectroscopy (AFS) [6, 16, 17], inductively coupled plasma optical emission spectrometry (ICP-OES) [18] and inductively coupled plasma mass spectrometry (ICP-MS) [19–23]. Among the techniques, ICP-MS could be considered as the most sensitive for the determination of trace metals in natural waters [24, 25]. On the other side, direct introduction of seawater samples to the plasma results in salt deposition on the ICP injector or walls of the torch tubes, or on the sampler/skimmer cones, reducing the analyte transport efficiency. Different approaches have been developed to overcome this problem [11, 19, 23, 26]. However, they can be susceptible to contamination problems and are time consuming. Cold vapour generation (CV) can separate mercury from complex matrices, minimizing non-spectral interferences and enhancing the analyte transport efficiency [17, 27].

Simplicity and reliability are the main properties required to the in-line methods applied in the process control of a candidate seawater certified reference material. This work describes the development and validation of a procedure for routine determination of mercury in seawater, at concentrations lower than the EQS, for the process control, stability and homogeneity assessment of a candidate CRM. Repeatability and intermediate precision of a method are the main factors for the selection of a method for homogeneity and stability studies of candidate CRMs. Both parameters allow to assess whether or not the observed variations between measurement results are in-line with the expected variation of the measurements over the processing sequence or time. In the validation of the method, the following parameters were assessed: linearity and working range, limit of detection (LOD) and limit of quantification (LOQ), trueness, repeatability and reproducibility and robustness. The individual uncertainty contributions of each parameter and the final expanded uncertainties have been estimated. The developed method could not only be easily applied for the CRM process control but also by control laboratories responsible for the mandatory chemical monitoring prescribed to the EU member states under the WFD.

## Experimental

### Reagents and certified reference material

A coastal seawater CRM, BCR-579, was provided by the European Commission, Joint Research Centre, Institute for Reference Materials and Measurements (IRMM, Geel, Belgium). The certified mercury mass fraction for this material is 1.9 ± 0.5 ng/kg.

Ultrapure water was supplied by the three-step ion exchange system, Milli-Q, fed by the reverse osmosis system, Elix 3, both from Millipore (El Paso, TX, USA). Sixty-five percent of ultrapure nitric acid (Merck KGaA, Darmstadt, Germany) was used for the preparation of acid matrices and cleaning solutions. Sodium tetrahydroborate (proanalysis grade; Merck KGaA, Darmstadt, Germany) and sodium hydroxide from Fluka (Sigma-Aldrich, St. Louis, MO, USA) were used as reductant agents. Sodium chloride for analysis (Merck KGaA, Darmstadt, Germany) was used to prepare artificial seawater matrices. Mercury solutions were prepared by diluting a 1000 mg/L certified solution (Merck KGaA, Darmstadt, Germany).

### Instrumentation

All measurements were conducted using a 7500CE inductively coupled plasma mass spectrometer (Agilent Technologies, Kobe-Shi Hyogo, Japan) equipped with a nickel sampler and skimmer cones. A HGX-200 advanced membrane cold vapour and hydride generation system (Cetac Technologies, Omaha, Nebraska, USA) was employed as a sample introduction system. Argon was added at two different steps: at the top of the gas–liquid separator, *carrier gas*, and after the PTFE membrane, *additional gas*. The additional gas allows a minimization of wash-out time and signal noise.

### Calibrant preparation

As salinity of the seawater can affect the efficiency of the reduction reaction, matrix matching was applied to calibrant and sample preparation. Calibration solutions were gravimetrically prepared using BCR-579 as matrix.

### Sample preparation

To preserve the dissolved mercury in seawater and avoid its loss by reduction [28], samples were acidified by adding 3 mL of ultrapure concentrated HNO_3_ per 40 mL of sample.

## Results

According to the ISO Guide 34 (http://www.iso.org/iso/home.html), validated analytical methods should be applied to characterize the reference material, during its development and production. Therefore, complete in-house validation and uncertainty estimation according to IUPAC [29] and EURACHEM [30] guidelines were carried out.

### Linearity and working range

A linear signal–mass fraction curve was found for the mercury determination. The calibration was performed using eight mercury mass fraction levels covering from 1.9 to 50 ng/kg. To counter potential memory effects, five replicates for each calibration solution were measured in a random order. Coefficients of determination (*R*^2^) higher than 0.9992 were obtained for 6 different calibration curves, and no outlying measurements >3 times the standard error of the calibration function were found.

### Limit of detection and limit of quantification

Ten independent replicate analyses of the BCR-579 were carried out under repeatability conditions. The LOD and LOQ were estimated as three or ten times the total standard deviation (*s*_T_), respectively. Total standard deviation includes the standard deviation of all the measurements (*s*_M_) and the standard deviation coming for the CRM. Equation 1 was applied to calculate the *s*_T_ value, where *s*_CRM_ is equal to 0.25 ng/kg. The LOD and LOQ values were 0.8 and 2.7 ng/kg, respectively. The LOQ value obtained is 13.5 % of the target value for the mass fraction of mercury in the candidate CRM (20 ng/kg). Moreover, the LOQ obtained is 8 times lower than the minimum performance criteria established by the Directive 2009/90/EC [3], according to which the LOQ of the methods used in the chemical monitoring program in the WFD should be equal or below a value of 30 % of the EQS.

1 $$ {s}_{\mathrm{T}}=\sqrt{S_{\mathrm{M}}^2+{S}_{\mathrm{CRM}}^2} $$

### Trueness

As there is no seawater certified reference material for mercury at the targeted mass fraction, the trueness was evaluated by gravimetrically spiking test portions of BCR-579 at two different levels, half and double of the targeted mass fraction, 10 and 40 ng/kg. Trueness was assessed by measuring three replicate samples on two different days. A recovery rate was calculated as the ratio between the found and the spiked concentration value. The mean recovery rates were 100.2 and 100.8 % for 10 and 40 ng/kg, respectively. The relative standard deviation in both cases was around 0.6 %.

### Repeatability and intermediate precision

Three sub-samples of a spiked sample were measured on five different days, using five different calibration curves. On each day, BCR-579 was gravimetrically spiked to obtain a final mercury mass fraction of 25 ng/kg. One-way ANOVA was used to estimate the repeatability and intermediate precision as within-group and between-group standard deviation, respectively (Table 1). The repeatability of the method (RSD_rep_) was 0.5 %, whereas the intermediate precision was 2.3 %.

**Table 1:** Mean of the mercury mass fraction (ng/kg) obtained from the repeatability and intermediate precision study

	Day 1	Day 2	Day 3	Day 4	Day 5
Replicate 1	24.41	25.31	24.50	24.30	25.60
Replicate 2	24.45	25.52	24.44	24.53	25.59
Replicate 3	24.53	25.44	24.44	24.74	25.68
Mean (ng/kg)	24.46	25.42	24.46	24.52	25.62
*s* (ng/kg)	0.06	0.10	0.03	0.22	0.05

### Robustness

The effect of the sample salinity on measurements was selected to evaluate the robustness of the method. Eight artificial seawater matrices were prepared using NaCl. The salinity ranged from 1.5 to 4.75 %. BCR-579 (with a salinity of 2.8 %) was taken as a reference matrix. The eight artificial seawater matrices and BCR-579 were spiked with the same mercury mass fraction, 15 ng/kg. Signals were measured for the spiked samples and for the blanks. The difference between the artificial seawater and the BCR-579 signals was evaluated by means of Eq. 2, where *I*_Spiked_ is the signal measured for the spiked sample, *I*_Blank_ is the mercury signal measured for the same salinity sample without spiking, and *C*_*i*_ is the amount of mercury added to the spiked sample. The subscript ‘*i*’ refers to the artificial seawater matrices prepared.

2 $$ {I}_i=\frac{i_{\mathrm{spiked}}-{i}_{\mathrm{blank}}}{C_i} $$

A regression analysis was made for the mercury signal against salinity. The slope was not significantly different from zero at the 99 % confidence level within the salinity range 1.5–4.75 %. However, a slight decrease on the signal was observed for the highest salinity (4.75 %). Student’s *t* test was applied to compare the mean of the results with the result obtained for the highest salinity. The obtained result showed that there is a statically difference between both values. Thus, the method was demonstrated to be robust for measurement of seawater samples with salinity in the range 1.5–4.5 %.

### Uncertainty estimation

The relative standard uncertainty contributions related to the repeatability and intermediate precision were obtained by applying one-way ANOVA to the 15 measurements. The following equations were applied:

3 $$ {u}_{\mathrm{rep}}=\sqrt{\frac{{\mathrm{RSD}}_{\mathrm{rep}}^2}{n_{\mathrm{rep}}}} $$

4 $$ {u}_{\mathrm{ip}}=\sqrt{\frac{{\mathrm{RSD}}_{\mathrm{ip}}^2}{n_{\mathrm{days}}}} $$

where RSD_rep_ is the repeatability of the method, *n*_rep_ is the number of replicates, RSD_ip_ is the intermediate precision, and *n*_days_ is the number of days. As Table 2 indicates, the relative standard uncertainty contributions were 0.13 and 1.03 % for the repeatability and the intermediate precision, respectively.

**Table 2:** Relative standard uncertainty contributions, equations applied and expanded relative uncertainty for measurement of mercury mass fraction in the seawater samples

	Relative standard uncertainty contribution (%)
*u*_ip_	1.03
*u*_rep_	0.13
*u*_t_	2.75
Expanded relative uncertainty (*U*, *k* = 2)	5.87

The relative standard uncertainty related to the trueness contribution was estimated by applying Eq. 5.

5 $$ {u}_{b,{x}_{\mathrm{ng}/\mathrm{kg}}}=\sqrt{2\frac{s^2}{n}} $$

where *s* is the standard deviation of the measurements and *n* is the number of measurements.

The relative standard trueness uncertainty was calculated at both spiking levels. The contributions obtained were 2.7 and 0.8 % at 10 and 40 ng/kg, respectively. As Table 2 shows, the highest contribution was considered as the contribution of the trueness relative standard uncertainty related to the expanded uncertainty.

The contributions of the repeatability, intermediate precision and trueness were taken into account for the calculation of the expanded uncertainty (*U*) of the measurements. The following equation was applied:

6 $$ U=k\cdot \sqrt{u_{\mathrm{rep}}^2+{u}_{\mathrm{ip}}^2+{u}_t^2} $$

where *U* is the expanded relative uncertainty, *k* is the coverage factor (*k* = 2), *u*_rep_ is the relative standard uncertainty of repeatability, *u*_ip_ is the relative standard uncertainty of intermediate precision, and *u*_t_ is the relative standard uncertainty of trueness. The coverage factor applied was 2 corresponding to the 95 % confidence level [31]. The final expanded uncertainty value is 5.87 %. The major source of uncertainty contribution is related to the trueness assessment.

### Process control of the candidate CRM

**Fig. 1:**
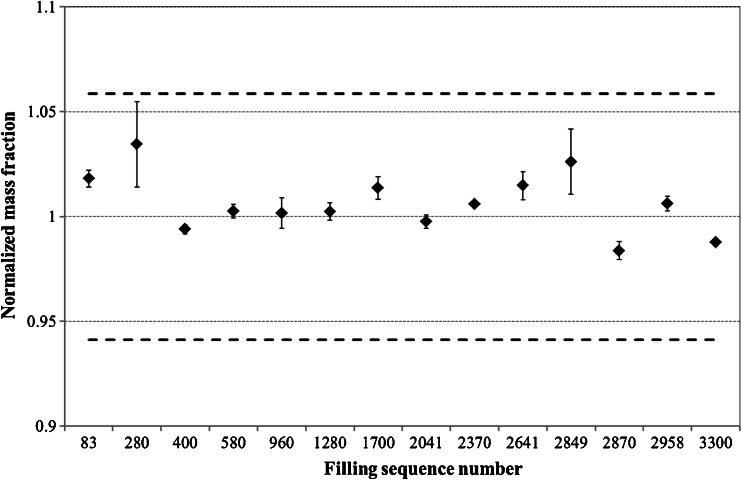
Normalized mercury mass fraction along the processing sequence of the material. The filled diamonds show the average value of three replicates of the same sample, the error bars correspond to the standard deviation of the three replicates

Considering the low level of mercury in the candidate certified reference material, in-line process control was considered essential and the filling procedure was continuously checked for signs of contamination. One ampoule was taken from the processing chain every 2 or 3 h, and the mercury mass fraction was determined. As shown in Fig. 1, the mass fraction measured was normalized to the targeted one (20.0 ng/kg). Control upper/lower limits were chosen, taking into account the uncertainty of the method, 1.00 ± 0.06 ng/kg (dotted lines).

### Homogeneity assessment of the candidate CRM

**Fig. 2:**
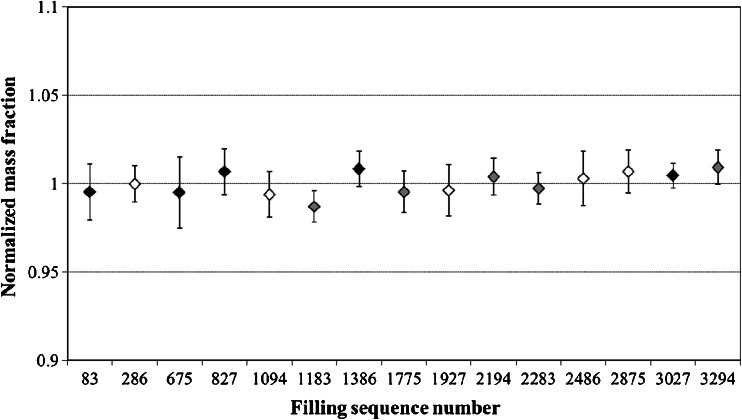
Normalized mercury mass fraction along the homogeneity study of the certification exercise of the candidate CRM. *Black results* were obtained the first day of measurements, *grey ones* the second day, and *white data* the third day

A key requirement for any reference material is the equivalence between the various units. Therefore, a between-unit homogeneity study was conducted with 6 replicate measurements made on each of 15 units. Measurements were performed in a randomized block design. However, all units could not be included in a single run due to time constraints. The maximum measurement time was set at 10 h, and one quality control sample was introduced in the analytical sequence every 1.5 h. Variations in measured mass fractions of 2 and 5.5 % were observed for a 25 ng kg^−1^ sample after 3 and 10 h, respectively. As indicated in Fig. 2, the mercury mass fraction measured is not affected by a run effect. Variances in the mass fraction determined are covered by the uncertainty related to the repeatability and intermediate precision.

## Conclusions

This paper presents a method that allows routine mercury quantification in seawater matrices at 30 % of the EQS level (20 ng/kg). The result fulfils the requirements of EU Directive 2009/90/EC with regard to uncertainty and LOQ. Moreover, the protocols for the treatment of the sample and the calibration solution are simple. Therefore, the developed method will be used for the CRM process control, but it can also be applied by control laboratories responsible for the chemical monitoring set under the WFD.
